# Influence of Non-Standard Tourist Accommodation’s Environmental Stimuli on Customer Loyalty: The Mediating Effect of Emotional Experience and the Moderating Effect of Personality Traits

**DOI:** 10.3390/ijerph19159671

**Published:** 2022-08-05

**Authors:** Shuzhen Liu, Xueji Wang, Lei Wang, Zhaoling Pang

**Affiliations:** 1 Joint Institute of Ningbo University and University of Angers, Ningbo University, Ningbo 315211, China; 2 Donghai Academy, Ningbo University, Ningbo 315211, China

**Keywords:** multi-dimensional environment of homestays, customer loyalty, emotional experience, Lijiang, S-O-R model

## Abstract

In recent years, minsu (homestays), a non-standard tourist accommodation, have been gaining popularity in China. The minsu has become an innovative driving force promoting the transformation of tourist destinations. This paper attempts to explore the relationship between the minsu’s environment (ambience) and customer loyalty, as customer loyalty is crucial to the sustainability of minsu and tourism destinations. This paper adopts Lijiang, China, as an empirical example to explore the relationship between the multi-dimensional environment of minsu and its influence on customer loyalty. Findings include: (1) the multi-dimensional environment perception of minsu includes spatial, cultural and social environment perception, all of which have a significant positive impact on emotional experience in varying degrees; (2) the emotional experience of minsu guests plays a significant mediating role between the minsu’s environmental perception and loyalty; (3) personality traits of openness, agreeableness and conscientiousness play a moderating role in the impact of residential space environment perception on emotional experience. Using the S-O-R model, this paper introduces emotional experience and personality traits into the relationship model between the minsu’s environment perception and customer loyalty and contributes to extant literature on the influence of customer loyalty in tourist destinations. The theoretical and practical implications are discussed.

## 1. Introduction

There is a growing trend within the tourism industry to transform from being a provider of mass sightseeing services to personalised, curated, high-quality tourist services [1]. In China, an alternative tourist accommodation known locally as minsu (homestays) has developed rapidly in recent years [2], according to the “China Tourism Minsu Development Report (2019)”, from 2016 to 2019, the number of minsu in mainland China increased from 50,000 to 169,000, a growth rate of 217.06% [3]. The proliferation of the minsu (otherwise known as homestay) reflects a buoyant tourist demand as such local-centric accommodation satisfies tourists’ desire for authentic experiences [4]. In many of China’s established and mature tourist destinations (e.g., the old town of Lijiang, Yunnan Province), the tourist accommodation industry has shifted towards providing minsu accommodation, with localised characteristics favoured by tourists.

A departure from traditional tourist accommodation such as hotels and guesthouses, minsu (note that plurality will not be regarded for this term in this paper given that it is a Chinese descriptor) emphasizes the host-guest experience in a creative living space that portrays the integration between local culture and tourism [5]. The minsu is usually designed with local flair that reflects local cultural norms in architecture style, spatial design and other local characteristics and has become a magnet for tourists keen to experience the authentic local cultures and customs in the tourist destination [6,7]. Minsu guests often desire social interaction, local cultural experiences, rest and relaxation and aesthetically pleasing accommodation [8,9,10,11]. The creativity of minsu and the differences in the desires of their guests are important factors that affect tourist experiences and distinguish the minsu from the typical tourist accommodation [12]. Extant literature has highlighted that ambient factors such as atmosphere, interior decoration and design, building style, amenities and social environment are important pull factors for attracting guests [13]. Ogucha et al. [14]’s research on homestay tourists’ satisfaction level uncovered that in the absence of tangible factors such as good service quality, the homestay must have other adequate tangible facilities to ensure repeat business and positive word-of-mouth recommendations. Gunasekarbtan et al. [15] pointed out that a homely ambience, value for money, local cultural experience and host-guest relationships are influential factors attracting guests to homestay accommodation. Previous studies on places of consumption have tended to focus on the ambience of shopping malls, restaurants and star-rated hotels [16,17,18]. They have not investigated the emergence of highly personalised accommodation spaces that emphasise meeting multiple tourists’ needs and are intertwined with the local characteristics of tourist destinations. To fill the current research gap, this paper attempts to pay attention to the significance of the minsu’s ambience and the resulting impact on the behaviour and emotional experience of minsu guests.

Studies have shown that servicescapes affect customers’ mood. Liu et al. [18] demonstrated the positive relationship between a good dining ambience and customers’ emotions while Ryu et al. [19] suggested that ambient factors affect consumers’ moods and their consumption behaviour. Theory on personality traits is often used to explain individual differences in emotion and behaviour. Dong [20] uncovered that university students with different personality types have different degrees of awe and happiness levels, while Qiu [21] suggested that personality traits have a moderating effect on the relationship between motivation and identity, which in turn affect behaviour. For minsu with its unique selling points of providing personalised and differentiated experiences, their guests’ personality traits and motivations are probably more distinct than guests of general, standard accommodation such as hotels. Taking this into account, it is worth exploring whether personality traits and personality types have a more substantial influence on the emotional experience of minsu guests. Therefore, this paper will explore whether personality traits have a moderating effect on guests’ perception on the minsu’s environment and emotional experience.

**Figure 1:**
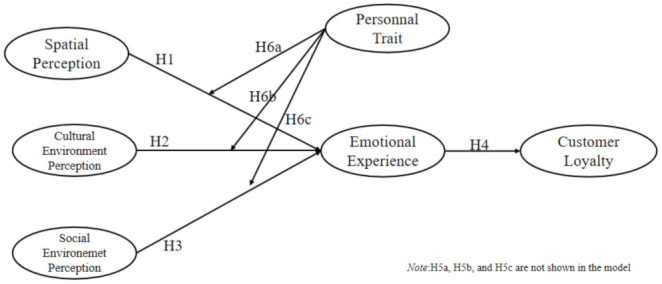
Conceptual model.

The S-O-R model is widely used to explore the relationship between the physical environment, customer’s emotions and customer’s behaviours. It is a popular mode frequently applied in hospitality studies [19,22,23]. In a similar vein, taking the S-O-R model as a conceptual framework, this paper attempts to construct a model on the relationship between the minsu’s multi-dimensional environment in tourism destinations (stimulus), customer’s experience (organism) and customer loyalty (response) [see Figure 1]. It attempts to shed light on how ambient factors in minsu affect guests’ emotions and loyalty, with emotions taking on a meditating role. By proposing measurement elements and indicators that influence the ambient factors of minsu, this study contributes to research on tourism accommodation by filling the existing research gap on the relationship between customer emotional experience and environmental perception. In an environment with limited provision of services, the minsu’s environmental perception is an important factor affecting guest perception and behaviour. By exploring the influence of different environmental factors on customers’ emotions and loyalty and providing practical suggestions for the investment, design and management of minsu accommodation, this study contributes pragmatically towards sustainable tourism development.

## 2. Literature Review

### 2.1. Environmental Perception

In recent years, China’s tourism industry has flourished. According to the “Basic Situation of Tourism Market in 2019” released by the China Tourism Academy (Research Center of the Ministry of Culture and Tourism), the total tourism revenue in 2019 reached 6.63 trillion yuan, an increase of 11% year-on-year. The total contribution of tourism to GDP is 10.94 trillion yuan, accounting for 11.05% of the total GDP. The rapid development of the tourism industry has resulted in constant changes in the demands of the tourism markets and consumers [24,25], resulting in differentiation in tourism accommodation. Against the backdrop of these changes, the minsu, with its unique selling point of “accommodation with warmth and soul”, has become a popular form of tourism accommodation for both hosts and guests. Sensing the growing importance of the nascent minsu tourism industry, the Chinese government has also started to support its growth. The minsu is regarded as a heart-warming post,-modern tourist accommodation with a homely ambience, exclusivity and warm host-guest relationships [26]. The study of the minsu is an important emergent research issue [27] as its differentiated characteristics and accommodation concepts have blurred the current boundaries of tourism accommodation [28]. Presently, the academic research on minsu is severely lagging behind the industry growth. Additionally, given the minsu’s complicated and unique features, unlike that of standard tourist accommodation, many minsu’s owners (known as hosts) face operation/management issues while the administrative departments of tourism destinations are still in the early exploratory stage of minsu management. To contribute toward extant literature on minsu, this study focuses on the minsu’s innovative development model by paying attention to its unique ambient factors while exploring minsu guests’ perception and its resulting impact in order to provide an in-depth understanding of this nascent business model and offer practical suggestions for practitioners of the minsu industry.

In July 2019, the Ministry of Culture and Tourism of China issued guidelines on the Basic Requirements and Evaluation of Minsu, which defines minsu as “small-scale accommodation facilities for tourists to experience local ecology, culture, economy and lifestyle by utilizing idling local resources such as vacant residential buildings, with no more than four floors of accommodation with a total building area restricted to 800 m^2^. The minsu’s hosts must be involved in guest reception services”. This study also adopts this official definition to define minsu from other forms of tourism accommodation. From this definition, it can be seen that there are special requirements to operate a minsu and the experience it provides to guests. Additionally, studies have demonstrated the importance of the minsu’s environment. Creating a satisfactory homestay atmosphere and providing an ideal environment for guests can give rise to customer loyalty [12]. There is a positive correlation between the minsu’s environment/ambience and consumer purchase intention, with a warm and friendly and sociable interactive environment significantly improving consumer purchase intention [29]. The host-guest relationship, the minsu’s natural, cultural ambience, and interior decoration are all significant pull factors for guests’ revisiting intention [30]. Therefore, it is significant to study the implications of the minsu’s particular environment and its resulting impact on customer loyalty.

The environmental design of the minsu often incorporates localised cultural features of the tourist destination. Tourists are attracted to stay in minsu because they desire to be more deeply immersed in the local culture [31]. Lijiang, a heritage tourist destination, is no exception to this phenomenon as Lijiang’s local minsu include distinctive elements of Lijiang’s unique culture. Based on extant research, the environmental surroundings play a significant role in the tourist experience. The servicescape of restaurants’ dining areas, such as decoration, architecture, colour, theme and facilities, play a determining role in influencing customers’ moods [32]. In minsu, ambient factors such as temperature, spatial design, interior décor, and colour schemes are predominantly guest-oriented. Long et al. [33] discovered that guests are more inclined to stay in homestays because the excellent interior decoration and facilities provide guests with a cosy and homely experience compared to standard hotel accommodation. The social environment, including the minsu’s host-guest relationship, also constitutes an important aspect of the minsu’s ambience [34]. In related research on Lijiang’s minsu, guests are attracted to these minsu because of the warm socialisation between the minsu host (boss), staff (housekeeper) and other minsu guests [13]. Therefore, the presence of warm host-guest relationships in minsu is a distinguishing factor from the standard hotel accommodation [5]. Wang et al. [35] highlighted that guests’ perspective of minsu is affected by the minsu’s cultural environment, distinguishing characteristics and the host-guest relationship dynamics. Considering the current research findings, this paper will differentiate the minsu’s ambience into three dimensions, namely, spatial, cultural, and social.

As an important tourist destination within China, Lijiang has a long history of minsu development. Additionally, given its status as a world heritage tourist destination, its minsu has significant differentiating characteristics in terms of architecture and spatial environment. Scholars such as Sun [13] have pointed out the unique local cultural features of Lijiang’s minsu ambient factors in terms of architecture, spatial design, customer service and so on, all of which are important pull factors for minsu guests. Xu [11] in his study on the Lijiang minsu’s experience divided the minsu experience into two aspects: aesthetic experience (cultural experience) and secular experience (environment and social experiences) and claimed that the overall minsu experience is influenced by these three factors. Within Lijiang, the spatial design and cultural environment of minsu are important aspects affecting the attractiveness of Lijiang as a tourist destination. How the minsu experience affects customer loyalty has a direct and significant influence on the sustainable development of the tourist destination too.

### 2.2. Emotional Experience

Emotional experience refers to how the individual responds to emotionally-induced situations through physiological and psychological behaviour [36]. The topic of emotional experience has become a popular topic in tourism research [36,37], and researchers have studied the impact of tourists’ emotional experience on customer satisfaction, loyalty and behavioural intention (i.e., the outcomes of emotional experience). Su et al. [38]’s research on China’s natural heritage tourism showed that tourists’ consumption intention affects their willingness for return visits. In contrast, Jani et al. [39] in their study on star-rated hotels, pointed out that customers’ positive and negative emotions directly affect the customer’s sense of loyalty. A burgeoning of research has also focused on the antecedents of tourists’ emotional experience, such as the factors affecting or inducing emotional experience. Additionally, environmental and other factors can trigger guests’ emotions, and these triggered emotions directly influence their behaviour intention [40]. Su et al. [38] further pointed out that the perceived service fairness in natural heritage tourism sites can trigger tourists’ positive or negative emotions; Tian et al. [41], in his research on mountain tourism destinations, uncovered that the natural environment with a religious atmosphere could induce tourists’ sense of awe. These studies on the antecedents and consequences of emotional experience prove that emotional experience can be used as a mediating variable to examine the impact mechanism of tourists’ behavioural intentions, such as customer loyalty. Therefore, this study will explore the mediating role of emotional experience in the relationship between the minsu’s environmental perception and customer loyalty.

### 2.3. Customer Loyalty

The concept of customer loyalty is often studied in marketing research. Oliver [42] defines loyalty as “a deeply held commitment to rebuy or repatronize a preferred product/service consistently in the future, thereby causing repetitive same-brand or samebrand-set purchasing, despite situational influences and marketing efforts having the potential to cause switching behavior”. Customer loyalty has the potential to increase market share and improves business profitability through customers’ repeated purchases [43]. In the same vein, customer loyalty is always a cornerstone of hospitality research. As WOM recommendations grow in importance, some researchers have started to regard WOM recommendations as a form of customer loyalty [18,44]. In tourism studies, the tourist’s intention to revisit is often considered largely similar to the concept of customer loyalty [45]. This paper defines the tourist’s customer loyalty as the tourist’s intention to revisit or willingness to provide WOM recommendations for the business establishment. Previous studies on customer loyalty tend to skew towards the relationship between satisfaction, perceived value and loyalty [46,47,48]. But some scholars, such as Babin et al. [16], highlighted that a good store atmosphere could stimulate the positive emotions of consumers, create customer value, and lead to customers’ willingness to return; Tian et al. [41] showed that a natural environment with religious atmosphere could induce the awe of tourists, promote WOM recommendation and enhance their willingness to revisit through customer satisfaction. As the minsu is a small-scale establishment compared to general star-rated hotels, it often faces manpower and insufficient marketing issues. Resultantly, most minsu rely on WOM recommendations or repeat customer visits for business continuity, highlighting the critical importance of customer loyalty. Therefore, this study aims to explore the determining factors that influence minsu’s customer loyalty.

## 3. Model and Hypotheses Development

### 3.1. The S-O-R Framework

This study adopted the “stimulus- organism-response” model that Mehrabian et al. [49] developed. Based on the S-O-R model, when an organism is exposed to an environment’s stimulus, its internal process, including its emotional and cognitive states, are changed, resulting in the organism making a response eventually. The external environment’s stimuli include tangible environment stimuli and intangible social environment stimuli [22]. The internal cognitive state refers to the emotional and cognitive states generated by individuals in psychology [50]. An individual response generally emphasises individual behavioural aspects of response.

The S-O-R framework has been widely adopted in the field of tourism and hospitality services. Su et al. [50] used the S-O-R model to show that consumption emotions and tourist destination identification are mediating factors between the tourist’s perceived tourist destination’s social responsibility and the pro-environmental behaviour of tourists. In their model, social responsibility is regarded as an external environmental stimulus, destination’s identity and consumption emotions as the internal emotional and cognitive states of the body, while the tourist’s environmental responsibility behaviour is an individual behavioural response. Dedeoglu et al. [22] studied the hotel’s servicescape and the factors responsible for the customers’ behavioural intentions and found that the hotel’s servicescape is considered the environmental stimulus, while the hotel guests’ perceived value is regarded as the internal cognitive state of the body, whereas the WOM recommendation and revisiting intention are regarded as individual responses. The study also proposed that the servicescape significantly impacts customer perceived value, with the perceived value having a positive impact on behavioural intention. In Li et al.’s study [23] on the hotel servicescape’s impact on customer’s civil behaviour, he regarded hotel servicescape as external stimuli, customer’s perceived value and cooperation as internal emotional responses, while customer’s civil behaviour is considered as individual responses. He found that customer’s perceived value and customer’s cooperation play mediating roles in the impact of servicescape on customers’ civil behaviour. Considering these research findings, this study uses the S-O-R model to explain the impact of the perceived environment on customer behaviour in the minsu context, where the minsu guest’s perception of the minsu’s environment/ambience is considered as the stimulus (S), the minsu guest’s emotional experience as the internal emotional perception (O), and the resulting customer loyalty is considered as response (R).

### 3.2. Hypotheses

#### 3.2.1. The Relationship between Minsu’s Environment Perception and Customer’s Emotional Experience

As mentioned previously, the spatial environment has an impact on customer emotional experience. Existing studies on shopping space have pointed out that creating a good spatial environment can evoke shoppers’ positive emotions, create customer value, and lead to repurchasing intention [16,17]. In tourism research, the natural environment of tourist attractions will also trigger specific emotions [41,51]. Environmental characteristics will also influence customers’ emotions significantly. Lijiang’s minsu are decorated distinctively in the local Nanxi style, with the minsu’s interior layout and decoration also reflecting personalised cultural style [13]. Some studies have pointed out that a rich cultural ambience can cultivate tourists’ awe experience, a finding confirmed in religious tourism [51] and red tourism [52] studies. In addition, social factors such as the service attitude and clothing of service staff and other customers will also influence customers’ perceptions and emotions; correspondingly, the relationship between customers and service personnel will affect the customer’s emotions [53]. It can be seen that social environmental factors will affect customers’ emotions and behaviours [54]. Therefore, based on the above, this paper proposes the following hypotheses:

**Hypothesis** **1** **(H1):** *The perception of the spatial environment of minsu has a significant positive impact on customers’ emotional experience*.

**Hypothesis** **2** **(H2):** *The perception of the cultural environment of minsu has a significant positive impact on customers’ emotional experience*.

**Hypothesis** **3** **(H3):** *The perception of the social interaction environment of minsu has a significant positive impact on customers’ emotional experience*.

#### 3.2.2. The Relationship between Emotional Experience and Customer Loyalty

Mehrabian et al. [49] pointed out in the S-O-R model that the response of the organism will affect the behaviour, in which the response of the organism includes emotional response. The resultant behaviour may be interpreted as approach behaviour or avoidant behaviour. The emotional state of customers influences their evaluation of consumption experience [54]. Dick et al. [55] highlighted that customers are more loyal after experiencing specific emotional reactions, while Sherman et al. [56] found a strong relationship between store customers’ purchasing behaviour and emotional experiences. Customers’ positive emotions in the servicescape are more likely to make them feel satisfied [57]. Within tourism studies, tourists’ loyalty is usually measured by revisiting intention, recommendation willingness and actual WOM recommendation [58]. Awe also exerts a positive influence on tourists’ WOM recommendation and revisiting intention [59]. When booking a stay at a minsu, guests are seeking functional, spiritual and cultural satisfaction and they will inevitably produce some form of negative or positive emotional reaction, leading to some behavioural changes. Additionally, positive emotions are considered to impact customer loyalty significantly [60]. Consequently, the following hypothesis is put forth:

**Hypothesis** **4** **(H4):** *The emotional experience of customers has a positive significant influence on customer loyalty*.

#### 3.2.3. The Mediating Role of Customer Emotional Experience

Studies have shown that environmental factors can trigger customers’ emotions and further affect their loyalty [55]. For minsu, the atmospheric spatial, cultural and social environment will trigger the customer’s emotional experience perception and then affect their behaviour. Correspondingly, the customer’s perception of the minsu’s atmosphere can trigger customer loyalty through emotional experience. Lee et al. [61] found that event tourists’ moods are moderated by environmental factors and affect their satisfaction level, including their willingness to provide WOM recommendations and revisiting intentions. Jang et al. [62] and Tian et al. [63] found that emotion plays a mediating role in environmental stimulation and customer behavioural intention and can trigger customer loyalty. Customers’ positive emotions play a mediating role in the relationship between minsu service and customer satisfaction [64]. Considering the above, this paper proposes the following hypotheses:

**Hypothesis** **5a** **(H5a):** *The emotional experience of minsu customers mediates the relationship between minsu spatial environment perception and customer loyalty*.

**Hypothesis** **5b** **(H5b):** *The emotional experience of minsu customers mediates the relationship between the perception of minsu cultural environment and customer loyalty*.

**Hypothesis** **5c** **(H5c):** *The emotional experience of minsu customers mediates the relationship between the social interaction environment and customer loyalty*.

#### 3.2.4. Personality Traits as Moderating Factor

Personality traits refer to an individual’s cognition, emotion and behavioural patterns, which are relatively stable and consistent [21]. The theoretical model of personality traits has been widely used in the field of psychological research, among which the ‘Big Five’ (i.e., extraversion, openness, agreeableness, conscientiousness and neuroticism) is the leading applied model [65]. Research findings have shown that extraversion is more related to positive emotions, while neuroticism is more related to negative emotions [39]. The onset of positive emotional tendencies differ among the ‘Big Five’ personalities (i.e., extraversion, conscientiousness, agreeableness, openness, and neuroticism) [66]. Dong [20] suggested some people experience awe more easily than others and suggested that individual personal differences account for the different degrees of awe experience. Qiu [21] shows that personality traits can moderate the relationship between civil servants’ customer service motivation and organisational identity, affecting their corporate organisational behaviour. When exploring the influence of personality traits in the S-O-R model, Jani et al. [67] found that personality traits play a moderating role in the relationship between environmental stimulation and emotional response. Considering the above, the following hypotheses are put forth:

**Hypothesis** **6c** **(H6c):** *Personality traits have a moderating influence in the impact of spatial environment perception on customers’ emotional experience*.

**Hypothesis** **6c** **(H6c):** *Personality traits have a moderating influence in the impact of cultural environment perception on customers’ emotional experience*.

**Hypothesis** **6c** **(H6c):** *Personality traits have a moderating influence in the impact of social interaction environment perception on customers’ emotional experience*.

Based on the above theories and research findings and taking into account the hypotheses formed in this paper, a proposed theoretical model illustrating the impact of the multi-dimensional environmental perception on customer loyalty is shown in Figure 1.

## 4. Research Design

### 4.1. Questionnaire and Scale Design

Data for this study were collected through a self-administered questionnaire. The questionnaire was designed to capture four main aspects: demographic variables (e.g., age, gender, educational level, occupation and number of visits to Lijiang); perception of minsu ambience, including spatial perception (e.g., temperature, air quality, interior design); cultural perception (localised and cultural aspects of minsu’s guest activities, food and beverages, service quality); social environment perception (e.g., interactions between fellow minsu guests and minsu staff); customer loyalty (e.g., WOM recommendation and revisiting intention); tourists’ personality traits, based on the Big Five Personality Traits (i.e., openness, conscientiousness, extraversion, agreeableness and neuroticism). Building on Costa and McCrae [68]’s Revised Neuroticism Extraversion Openness Personality Inventory (NEO-PI-R), this study adopted a revised Big 5 personality traits modified by Chinese scholars for application in China’s cultural context [69,70].

As shown in Table 1, the questions of each variable were designed with reference to relevant literature, combined with the actual situation of the case, and revised and improved after the pre-survey. The questionnaire (except for emotional experience) was measured by the Likert 7-point system [“strongly disagree (1 point)” to “strongly agree (7 points)”]. The emotional experience was measured through semantic differences in the descriptive language used within the questionnaire.

### 4.2. Site Selection

Lijiang Old Town is located in Lijiang, Yunnan Province, China. It was built in the late Song Dynasty and the early Yuan Dynasty, with a history of more than 800 years. Lijiang is located at the crossroads of Yunnan, Sichuan, and Tibet provinces. It has been an important town along the ancient Tea-horse road since ancient times. Multiple cultures have been integrated and accumulated in Lijiang Old Town, forming a national culture represented by Naxi culture and Naxi business culture. Naxi dwellings, Dongba Culture, Naxi ancient music, etc., are the most typical representations of this national culture. In 1997, the Old Town of Lijiang was inscribed on the UNESCO World Heritage List, and the number of tourists rapidly increased to 1.73 million [73]. Since then, Lijiang’s tourism industry has thrived. By 2019, Lijiang had received a total of 54.0235 million tourists with a total tourism revenue of 107.826 billion Yuan. Since the early 1990s, tourism has been developed in the old town of Lijiang, with tourism activities gradually becoming an economic pillar in Lijiang’s growth. The rapid development of tourism has also spurred the rapid growth of Lijiang’s minsu. According to relevant statistics, there were 2843 minsu in Lijiang in 2016 [74], and as of 2019, the number had exceeded 6000 [3]. Lijiang’s minsu development and its guest reception services started early in Lijiang’s tourist development and is now one of the most distinctive cultural attractions within Lijiang. The Lijiang minsu industry is also one of the most popular business circles for domestic tourists within China. Many of Lijiang’s minsu feature strong local cultural characteristics as they are mainly housed within the local Naxi tribe’s residential buildings. Furthermore, Lijiang has a reputation as a ‘slow-living’ (leisurely) tourist destination [75,76], with tourists having a tendency to stay longer in Lijiang for a more authentic local experience. The minsu experience and social interaction with the minsu’s hosts and guests is also an integral part of the Lijiang tourist experience. Considering the above, the old town of Lijiang is a suitable reference study for the exploration of the minsu experience so as to provide insights for other tourist destinations with minsu.

### 4.3. Data Collection and Research Objects

The data for this study were collected through a questionnaire distributed to tourists with accommodation experience in Lijiang. In order to obtain a more abundant research sample, this study chose to carry out a questionnaire survey during the peak tourism season in Lijiang with offline data collection carried out in the ancient city of Lijiang from July 26 to 31. As shown in Table 2, the analysis of respondents’ demographics indicated that within the 563 respondents, 51.9% were male and 48.1% were female. With respect to age, about 56% of the total tourists were primarily concentrated between 19 and 29, and about 26.1% were primarily concentrated between 30 to 45 years. At the level of educational background, the results showed that the participants had a high level of education, with undergraduate or above degrees accounting for 71.9%. In terms of occupation, students made up most of the participants, accounting for 38.7%, followed by self-employed and business people, accounting for 15.6% and 14.7%, respectively. As for the number of times to visit Lijiang, more than half of the participants visited Lijiang for the first time (32.5%), and the second time (34.6%); while, 51.3% went with family members and 40.9% with friends. Finally, participants who plan to stay in Lijiang for 3–5 days account for 49.6%, and those who plan to stay for 1–2 days account for 28.8%. The overall structure of the questionnaire is good, which is conducive to the universality of the analysis results.

## 5. Data Analysis and Research Findings

### 5.1. Reliability and Validity Analysis

In order to ensure the validity of model-fitting degree evaluation and hypotheses testing, this paper uses SPSS26.0 for reliability analysis. Cronbach’s Alpha reliability coefficient is used to check the consistency degree of research variables on each measurement item, and its value is between 0 and 1. The higher the value of α is, the better the correlation between questionnaire items, and the higher the internal consistency reliability. As shown in Table 3, the α values of each latent variable are between 0.769–0.906 (αspatial perception = 0.896, αcultural environment perception = 0.906, αsocial environment perception = 0.769, αemotional experience = 0.886, αcustomer loyalty = 0.887), indicating that the variables have good internal consistency reliability.

The validity analysis of the survey included content validity and internal construct validity. Content validity refers to how well the survey’s content measures the construct that it is designed to measure. This study has designed the survey items based on knowledge gathered from extant literature and improvements made to the wording and expression of the survey items through small-scale testing and interviews to ensure the content validity of the final survey. After KMO value analysis and Bartlett sphericity test, the test results showed that KMO = 0.938 and Bartlett sphericity test results were significant (Sig. = 0.000), which were suitable for further factor analysis.

AMOS was used to test the internal structure validity of the scale. Internal structure validity refers to whether the data structure obtained with the measurement tool is consistent with the expectation of the construct. As shown in Table 3, the standard factor load of most of the observed variables > 0.7, *p* < 0.001, the fitting indexes of the model (χ^2^/DF = 3.891, RMSEA = 0.072, CFI = 0.919, RMR = 0.084, GFI = 0.895, NNFI = 0.908, NFI = 0.895) basically meet the evaluation criteria and meet the requirements [77], showing good convergence validity. Confirmatory factor analysis (CFA) was conducted on 24 observed variables. Five latent variables passed the reliability and validity test by using the maximum likelihood estimation method, and also calculated the composite reliability (CR) and average variance extracted (AVE). The results show that the CR values of the five latent variables were above 0.7, while the AVE values were below 0.5 except for social environment perception indicating that the latent variables exhibited good internal consistency and convergent validity. The results of the discriminant validity test are shown in Table 4. Sufficient discriminant validity for this study was also established as the squared correlation between a pair of variables is less than the AVE value of each individual variable.

### 5.2. Hypotheses Testing

**Figure 2:**
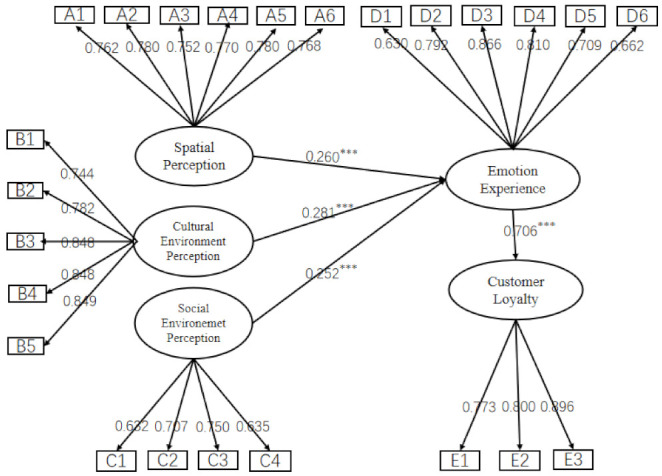
Test results. *** *p* < 0.001.

Figure 2 present the standardised path coefficients proposed in the model. It can be seen that customers’ emotional experience is influenced by customers’ perception of the spatial environment (β = 0.260, *p* < 0.001), cultural environment perception (β = 0.281, *p* < 0.001) and social environment perception (β = 0.252, *p* < 0.001), supporting Hypothesis 1, Hypothesis 2 and Hypothesis 3. These results indicate that cultural environment perception has the most influence on emotional experience (β = 0.281), followed by spatial environment perception (β = 0.260) and social interaction environment perception (β = 0.252). Meanwhile, customer emotional experience also had a significant positive impact on customer loyalty (β = 0.706, *p* < 0.001), establishing Hypothesis 4 as valid.

In order to test the mediating effect of emotional experience between environmental perception and customer loyalty, SPSS-Process Macro was used to analyse the mediating effect. A 5000-bootstrap sample with 95% confidence levels was used. If the CI contains zero, this indicates that there is no mediating effect. If the CI does not contain zero, this indicates that the mediating effect exists. Our results are shown in Table 5. The indirect effect does not contain zero, establishing H5a, H5b, and H5c as valid.

Under the assumption of the existence of a mediating effect, if the CI of the direct effect contains zero, it is considered a complete mediation effect. Otherwise, it is considered a partial mediation effect. From the data analysis, environmental perception indirectly influences customer loyalty through emotional experience. Similarly, emotional experience partially mediates between spatial environment perception and customer loyalty with its standardised indirect effect as 0.262 (95% CI as 0.202, 0.331). Emotional experience directly and partially mediates cultural environment perception and customer loyalty, and its standardised indirect effect was 0.202 (95% CI as 0.152, 0.256). Additionally, the relationship between the social environment and customer loyalty is also partially mediated by emotional experience with its standardised indirect effect as 0.218 (95% CI as 0.168, 0.273).

In this study, SPSS-Process Macro was used to examine the moderating effect of personality traits on the relationship between environmental perception and emotional experience. Table 6 and Table 7 show that the interaction between spatial environment perception and personality traits has a significant impact on emotional experience (*p* < 0.01). Results also show that the moderating effects of agreeableness (β = 0.167 **), conscientiousness(β = 0.145 **) and openness (β = 0.144 **) were significant; results were ranked in order based on the moderating effect. H6a is partially supported. Given that the relationship between cultural environment perception and personality traits has an insignificant influence on emotional experience (*p* > 0.05), demonstrating that personality traits do not play a moderating role in the influence of cultural environment perception on emotional experience. Assuming that H6b is not supported. The interaction between social environment perception and personality traits will have a significant impact on emotional experience (*p* < 0.01). Therefore, openness (β = 0.183 ***), conscientiousness (β = 0.180 ***) and neuroticism (β = 0.073 *) had a significant moderating effect (ranked in terms of the strength of the moderating effect) in which H6c was partially supported (see Table 8).

## 6. Conclusions

### 6.1. Conclusions and Discussion

Based on the S-O-R theoretical model, this paper constructs a multi-dimensional model taking into account environmental perception, customer emotional experience and customer loyalty (see Figure 1). This paper explores the differences in the impact of different minsu on customer emotions and the mediating role of customer emotions between environmental perception and loyalty and explores the moderating role of personality traits between environmental perception and emotional experience. Our research findings contribute towards a better understanding of the relationship between minsu and customer loyalty and expand extant knowledge on minsu research. Further, it adds to the related research on the relationship between customer emotional experience and the environment, thereby supplementing the existing research on tourism accommodation.

This study uses the S-O-R model to explore how the environment of Lijiang’s minsu affects customer loyalty. First, the relationship between customers’ perception of minsu and emotional experience was examined. It is found that external environmental stimuli have a significant impact on emotional experience, and minsu guests’ perception of the ambient factors of the minsu has a significant positive effect on guests’ emotional experience, further verifying that ambient factors exert a significant influence on customers’ emotions [32]. Additionally, guests’ perception of the minsu’s cultural environment has a positive correlation with their emotional experience, a finding which is congruent with Qi’s et al. [51] study, wherein religious and cultural atmosphere are found to have identical impacts on visitors’ emotions. Results also show that the perception of the minsu’s social environment significantly, positively impacts customers’ emotional experience. This research conclusion is in response to Liu et al.’s [64] research on the impact of social servicescape in minsu on customers’ positive emotions.

Next, this paper presents clear findings that indicate the emotional experience of minsu guests has a direct implication on customer loyalty. The stronger the emotional experience of minsu guests, the higher the customer loyalty. In addition, through our integrated framework, the emotional experience of minsu guests is verified to play a mediating role between minsu environmental perception (spatial environment, cultural environment perception, social environment perception) and customer loyalty. Both Jani et al. [39] and Ellen et al. [40] present similar findings indicating that a restaurant’s servicescape and ambient factors can affect customers’ emotional state and, subsequently, their behavioural intention.

Finally, the analysis of the moderating effect of personality traits shows that the openness, agreeableness and conscientiousness traits play a moderating role in the impact of the minsu’s spatial environment perception on emotional experience. Additionally, the openness, conscientiousness and neuroticism traits of minsu guests play a moderating role in the impact of residential social interaction environment perception on emotional experience. It is congruent with the cognitive appraisal theory of emotion, which postulates that personal emotions are generated by the interaction between people and the environment. Individuals are not passive recipients of external stimuli; we take active steps to moderate our response to the external stimuli [78], a finding consistent with Jani et al. [67]’s research on the relationship between personality, customer satisfaction, image, ambience and loyalty. This study better explains the impact of the multi-dimensional environment on customer emotional experience and loyalty, clarifies the influencing variables, furthers the understanding of the multi-dimensional environment and its significance, and enriches the existing research on the driving factors of minsu guests’ loyalty.

### 6.2. Contributions and Implications

First, studies on environmental perception in the field of tourism mainly focus on tourists’ or residents’ perceptions of relatively large-scale environments such as tourist destinations or scenic spots. These studies confirmed that environmental perception is intrinsically related to tourists’ emotions [79] and behavioural intentions [80]. However, few studies have explored the relationship between them in the field of tourism accommodation. Moreover, minsu is often overlooked even though it is an emerging non-standard accommodation that emphasizes the importance of the environment. This study applies environmental perception to the study of minsu and further segregates the environmental perception of minsu into three dimensions: spatial, cultural and social environmental perception, broadening the current research boundary on environmental perception. It not only deepens the understanding of the importance of the multi-dimensional environment of minsu but also enriches the related research on minsu and broadens the research fields related to environmental perception. Additionally, previous studies have neglected the mediating role of emotional experience between environmental perception and customer loyalty. By testing the mediating role of emotional experience, our research findings enrich the study of the relationship between external environmental stimuli and individual behavioural intention from the perspective of personal emotional experience. Moreover, this paper also examines the moderating effect of the ‘Big 5′ personality traits on the impact of minsu’s environmental perception on customers’ emotional experience, which is beneficial to advancing the understanding of the differences between individual emotional reactions and behaviours.

Subsequently, the relationship between minsu guest environmental perspective and customer loyalty is also verified. The emotional experience triggered by the environment will affect consumption behaviour [40]. Similarly, the minsu environment will affect customers’ emotional experience and then have a significant positive impact on customer loyalty. Therefore, minsu hosts should focus on their guests ‘emotional experience and create a good ambient environment for customers from the aspects of space design, culture and social interaction to encourage guests’ revisiting intentions or WOM recommendations. Specifically, hosts could build a comfortable and conducive ambient servicescape with a rich cultural atmosphere and provide customers with ample opportunities for socialisation (such as host-guest and guest-guest interactions) to promote a more positive emotional experience for their guests, strengthen the minsu guests’ attachment to the minsu, and cultivate their desire to revisit or to engage in WOM recommendations. This will help to realise the sustainable development of minsu and, resultantly, play a stronger role in promoting tourist destinations.

Finally, research findings also discovered that the minsu guest’s personality traits are a key moderating factor, given that customers with different personality traits will have different degrees of emotional experience in minsu. Therefore, their customer loyalty to the minsu will also differ. Minsu hosts could take the initiative to understand their customer’s personality traits through host-guest socialisation so as to provide targeted customer services and develop or design related products. To achieve this, minsu hosts have to strengthen their personal and professional development and improve their socialisation skills too.

To sum up, in the limited service situation, the minsu’s environment is an important factor affecting customer experience and behavioural intentions. Environmental dimensions such as space, culture, and society have different inherent impacts on the emotional experience, particularly the impact of cultural environment perception, which is the most significant. To some extent, this study can provide a reference for the investment, design, and operation management of minsu in the future, which is beneficial to promote the high-quality development of the minsu industry.

### 6.3. Limitations and Future Research Directions

The applicability of the theoretical model discussed in this paper remains to be tested. In this study, our theoretical model is empirically tested by using the old town of Lijiang’s minsu. Lijiang’s minsu has unique architectural characteristics, a rich local cultural atmosphere and a ‘slow-living’ pace of life, all of which encourage socialisation. Therefore, whether the findings can be generalised to other tourist destinations remains to be tested by future studies. Furthermore, this study focuses on how the minsu’s internal environment, such as spatial, cultural and social environment, affects customer loyalty. The larger environment of the tourist destination is not considered to a certain extent. Follow-up studies could consider exploring the impact of the tourist destination’s environment on customer loyalty. Finally, this study was carried out during the COVID-19 epidemic, which increased the difficulty of the research to some extent, especially the lack of sample data on foreign tourists. This limited the comparison of tourists’ behaviours from different cultural backgrounds. In addition, during the epidemic, the public has a strong awareness of maintaining social distance, which may affect the perception of the social interaction environment of minsu. In future, cross-cultural comparative research could be carried out on this topic.

## Figures and Tables

**Table 1:** Design of the Questionnaire.

Latent Variables	Questions	
Spatial Perception	A1. This minsu is clean and hygienic.	[71]
	A2. This minsu’s temperature is comfortable.	
	A3. This minsu’s air is fresh.	
	A4. This minsu’s design attracts me.	
	A5. This minsu’s interior design makes me comfortable	
	A6. This minsu’s colour theme makes me feel relaxed.	
Cultural Environment Perception	B1. This minsu’s activities allow me to experience local culture.	[71]
	B2. This minsu’s food is very localised.	
	B3. This minsu’s services reflect local culture.	
	B4. This minsu reflects the authentic lifestyle of locals.	
	B5. I can experience the history of this place in this minsu.	
Social Environment Perception	C1. In this minsu, I can interact with the other minsu’s guests.	[71,72]
	C2. This minus has enthusiastic service staff.	
	C3. This minsu’s guests are friendly.	
	C4. This minsu has good social spaces.	
Emotional Experience	D1. In this minsu, I feel (sleepy-excited).	[51,63]
	D2. The minsu’s accommodation experience is (normal-exceptional).	
	D3. The minsu’s accommodation experience is (ordinary-extraordinary).	
	D4. The minsu’s accommodation experience is (forgettable-unforgettable)	
	D5. In this minsu, I am treated with (contempt-respect)	
	D6. In this minsu, I feel (low-spirited-high-spirited).	
Customer Loyalty	E1. I will choose this minsu as my first choice the next time I visit Lijiang.	[47,71]
	E2. I am willing to recommend this minsu to others.	
	E3. I am willing to share my experience in this minsu with others.	
Openness	O1. I get very excited about new ideas.	[68,69,70]
	O2. I enjoy thinking.	
	O3. I enjoy accepting new ideas.	
	O4. I enjoy thinking about the deeper meanings of things.	
	O5. I am a very imaginative person.	
Conscientiousness	P6. I can carry out my plans very well.	
	P7. I am meticulous.	
	P8. I am always well-prepared.	
	P9. I can keep to the plans I made.	
	P10. I am very serious about my work.	
Extraversion	R11. I interact with many different people at parties.	
	R12. I feel very comfortable around people.	
	R13. I start conversations easily.	
	R14. I make friends easily.	
	R15. I do not mind being the centre of attention.	
Agreeableness	S16. I empathise with others’ feelings.	
	S17. I am concerned about others.	
	S18. I respect others.	
	S19. I believe that people around me have good intentions.	
	S20. I believe what others say to me.	
Neuroticism	T21. I worry easily about things.	
	T22. I am also easily worried.	
	T23. I always fear for the worst.	
	T24. I am always filled with doubts.	
	T25. I panic easily.	

**Table 2:** Descriptive statistics showing sample characteristics.

Varibles	Frequency	%	Varibles	Frequency	%
Gender			Number of visits to Lijiang		
Male	292	51.90	First time	183	32.50
Female	271	48.10	Second time	195	34.60
Age			Twice time	79	14.00
<18	79	14.00	Fourth time and above	106	18.90
19–29	315	56.00	A companion to Lijiang		
30–45	147	26.10	Friend	230	40.90
46–59	21	3.70	Family member	289	51.30
60 and above	1	0.20	Colleague	31	5.50
Education			Tour group	6	1.10
High school and below	158	28.10	Alone	7	1.20
Junior college	143	25.40	Number of days planned in Lijiang		
Undergraduate	205	36.40	1–2 days	162	28.80
Postgraduate and above	57	10.10	3–5 days	279	49.60
Occupational Status			6–10 days	66	11.70
Student	218	38.70	11–20 days	10	1.80
Government functionary	28	5.00	>21 days	46	8.20
Self-employed	88	15.60			
Teacher	52	9.20			
Businessman	83	14.70			
Else	94	16.70			

**Table 3:** Factor analysis.

	Questions	λ	Cronbach α	CR	AVE
Spatial Perception	A1. This minsu is clean and hygienic.	0.762	0.896	0.897	0.592
	A2. This minsu’s temperature is comfortable.	0.780			
	A3. This minsu’s air is fresh.	0.752			
	A4. This minsu’s design attracts me.	0.770			
	A5. This minsu’s interior design makes me comfortable.	0.780			
	A6. This minsu’s colour theme makes me feel relaxed.	0.768			
Cultural Environment Perception	B1. This minsu’s activities allow me to experience local culture.	0.744	0.906	0.907	0.663
	B2. This minsu’s food is very localised.	0.782			
	B3. This minsu’s services reflect local culture.	0.848			
	B4. This minsu reflects the authentic lifestyle of locals.	0.848			
	B5. I can experience the history of this place in this minsu.	0.849			
Social Environment Perception	C1. In this minsu, I can interact with the other minsu’s guests.	0.632	0.769	0.772	0.459
	C2. This minsu has enthusiastic service staff.	0.707			
	C3. This minsu’s guests are friendly.	0.75			
	C4. This minsu has good social spaces.	0.635			
Emotional Experience	D1. In this minsu, I feel (sleepy- excited).	0.630	0.886	0.892	0.59
	D2. The minsu’s accommodation experience is (normal-exceptional).	0.792			
	D3. The minsu’s accommodation experience is (ordinary-extraordinary).	0.866			
	D4. The minsu’s accommodation experience is (forgettable-unforgettable)	0.810			
	D5. In this minsu, I am treated with (contempt-respect)	0.709			
	D6. In this minsu, I feel (low-spirited-high-spirited).	0.662			
Customer Loyalty	E1. I will choose this minsu as my first choice the next time I visit Lijiang.	0.773	0.887	0.891	0.732
	E2. I am willing to recommend this minsu to others.	0.800			
	E3. I am willing to share my experience in this minsu with others.	0.896			

**Table 4:** Discriminant validity test.

Variables	Spatial Perception	CEP	SEP	Emotional Experience	Customer Loyalty
Spatial Perception	0.769				
CEP	0.508 **	0.814			
SEP	0.554 **	0.616 **	0.677		
Emotional Experience	0.487 **	0.517 **	0.507 **	0.768	
Customer Loyalty	0.608 **	0.633 **	0.578 **	0.613 **	0.856

The value on the diagonal is the square root of AVE, and the value below the diagonal is the correlation coefficient between variables. CEP = cultural environment perception, SEP = social environment perception. ** *p* < 0.01.

**Table 5:** Analysis of the mediation effect.

Path	Direct Effect	S.E.	95% Confidence Levels		Indirect Effect	S.E.	95% Confidence Levels	
			Lower	Upper			Lower	Upper
H5a SP—EE—CL	0.525	0.044	0.438	0.612	0.262	0.032	0.202	0.331
H5b CEP—EE—CL	0.431	0.034	0.363	0.499	0.202	0.026	0.152	0.256
H5c SEP—EE—CL	0.360	0.036	0.290	0.430	0.218	0.027	0.168	0.273

SP = Spatial Perception, CEP = Cultural environment perception, SEP = Social environment perception, EE = Emotional Experience, CL = Customer Loyalty.

**Table 6:** Analysis of moderating effect 1.

Hypothesis	Path	β	t	*p* Value
H 6a	SP × PT→EE	0.229	3.050	0.002
H 6b	CEP × PT→EE	0.045	0.791	0.430
H 6c	SEP × PT→EE	0.246	3.389	0.001

SP = Spatial Perception, CEP = cultural environment perception, SEP = social environment perception, EE = Emotional Experience, PT = personality traits.

**Table 7:** Analysis of moderating effect 2.

	PT	Openness	Agreeableness	conscientiousness	Extraversion	Neuroticism
EP						
SP→EE		β = 0.144 **	β = 0.167 **	β = 0.145 **	β = 0.125	β = 0.029
SEP→EE		β = 0.183 ***	β = 0.083	β = 0.180 ***	β = 0.027	β = 0.073 *

SP = Spatial Perception, SEP = social environment perception, EE = Emotional Experience, PT = personality traits. EP = Environment Perception. *** *p* < 0.001; ** *p* < 0.01; * *p* < 0.05.

**Table 8:** Results of hypothesis testing.

Hypothesised Path	Hypothesis
H1: Spatial Perception→Emotional Experience	Supported
H2: Cultural Environment Perception→Emotional Experience	Supported
H3: Social Environment Perception→Emotional Experience	Supported
H4: Emotional Experience→Customer Loyalty	Supported
H5a: Spatial Perception→Emotional Experience→Customer Loyalty	Supported
H5b: Cultural Environment Perception→Emotional Experience→Customer Loyalty	Supported
H5c: Social Environment Perception→Emotional Experience→Customer Loyalty	Supported
H6a: Spatial Perception × personality trait→Emotional Experience	Partly Supported
H6b: Cultural Environment Perception × personality trait→Emotional Experience	Not Supported
H6c: Social Environment Perception × personality trait→Emotional Experience	Partly Supported

## Data Availability

Not applicable.
